# Endothelial marker profiles in cerebral radiation-induced vasculopathy: A comparative immunohistochemical analysis

**DOI:** 10.1097/MD.0000000000037130

**Published:** 2023-02-02

**Authors:** Mohammad Mohsen Mosleh, Moon-Jun Sohn, Han Seong Kim

**Affiliations:** a Department of Biomedical Science, Graduate School of Medicine, Inje University, Busanjin-gu, Busan, Korea; b Department of Neurosurgery and Neuroscience & Radiosurgery Hybrid Research Center, Inje University Ilsan Paik Hospital, College of Medicine, Ilsanseo-gu, Goyang City, Gyeonggi-do, Korea; c Department of Pathology, Inje University Ilsan Paik Hospital, Inje University Ilsan Paik Hospital, College of Medicine, Ilsanseo-gu, Goyang City, Gyeonggi-do, Korea.

**Keywords:** endothelial cell injury, immunohistochemistry, radiation-induced vasculopathy, radiosurgery

## Abstract

Radiation therapy results in radiation-induced vasculopathy, characterized by alterations in the vascular architecture stemming from radiation exposure. The exact molecular pathways and associated pathologies of this condition have yet to be comprehensively understood. This study aimed to identify specific markers’ roles in cerebral vascular endothelial injury pathogenesis after radiosurgery and explore their unique expression patterns in diverse pathologies post-stereotactic radiosurgery. A retrospective cohort study was conducted to assess the expression profiles of endothelial markers via immunohistochemical analysis in 25 adult patients (13 males and 12 females) who had undergone neurosurgical resection for various central nervous system pathologies following stereotactic radiosurgery or radiotherapy from 2001 to 2015. Our findings revealed strong immunohistochemical expression of ICAM-1 and E-selectin across various disease states, while MMP-9, PAI-1, and eNOS exhibited moderate expression levels. In contrast, VCAM-1 and P-Selectin had the weakest expression across all groups. Notably, while individual markers showed significant variations in expression levels when comparing different diseases (*P* < .001), no substantial differences were found in the overall immunohistochemical expression patterns across the 5 distinct pathologies studied (*P* = .407, via 2-way ANOVA). Despite the varied long-term effects of radiotherapy on the vascular endothelium, a common thread of inflammation runs through the pathology of these conditions. The distinct patterns of marker expression identified in our study suggest that different markers play unique roles in the development of radiation-induced vasculopathy. These findings offer insights that could lead to the development of novel preventive strategies and treatments.

**Key Points**

Question: How is the immunohistochemical expression of cerebral vascular endothelial specific markers following radiosurgery/radiotherapy?

Findings: In this retrospective cohort study that included 25 adults, strong immunohistochemical expression of ICAM-1 and E-selectin was revealed across various disease states, while MMP-9, PAI-1, and eNOS exhibited moderate expression levels.

Meaning: Despite the varied long-term effects of radiotherapy on the vascular endothelium, a common thread of inflammation runs through the pathology of these conditions.

## 1. Introduction

Radiation vasculopathy, characterized by pathological reorganization of vascular tissue post-radiation exposure, is a significant concern, with radiation-induced cerebral vasculopathy reported in 17% to 93% of cases.^[1,2]^ Factors such as radiation dosage, exposure duration, and tissue type influence outcomes, and diagnosis latency can extend up to 25 years.^[1]^ Late vascular complications of cranial irradiation encompass diverse manifestations, including cerebrovascular accidents, lacunar lesions, and occlusive diseases.^[3]^ Recent insights suggest a multifaceted interaction between various brain cell populations leading to progressive neurological damage.^[4,5]^ The vascular endothelium, pivotal in cardiovascular homeostasis, is sensitive to radiation injury.^[6]^ Following radiation, initial vascular changes compromise the blood-brain barrier, leading to vasogenic edema and a hypoxic brain environment. Subsequent oxidative stress causes endothelial damage, impairing vasomotor response and endothelial structure. Acute phases may display thrombus formation and hemorrhage, while late phases exhibit capillary deterioration, scarring, and fibrosis.^[7]^ Endothelial dysfunction, resulting from various pathophysiological stimuli, including radiation, promotes an environment prone to inflammation, coagulation, and thrombosis.^[8–10]^ Following radiation injury to vascular endothelium, a cascade of molecular and cellular events ensue, including endothelial activation either directly through radiation or indirectly via reactive oxygen species (ROS). This cascade leads to inflammation, plaque formation, accelerated atherosclerosis, thrombosis, and fibrosis. Various pathways and markers, such as Chemoattractant protein-1 (MCP-1), intercellular adhesion molecule-1 (ICAM-1), P-selectin, E-selectin, vascular adhesion molecule-1 (VCAM-1), matrix metalloproteinase-9 (MMP-9), and inflammatory and growth factors contribute to immune cell attraction, transmigration, and initiation and protraction of inflammation.^[9,11,12]^ Leukocyte adhesion to the endothelium initiates the inflammatory phase of atherogenesis.^[13]^ The establishment and progression of atherosclerotic lesions involve inflammatory cytokines, vascular smooth muscle transformations, and leukocyte and apoptotic cell accumulation.^[14]^ Plasminogen activator inhibitor-1 (PAI-1) and Endothelial-to-Mesenchymal Transition further contribute to plaque development, leading to thrombosis.^[15]^ Neovascularization, a risk factor for plaque instability, can accelerate endothelial lesion progression, increase oxidative stress, and elevate inflammatory cell recruitment.^[16]^ Addressing long-term side effects post-radiotherapy, especially cerebral vasculopathy, is crucial due to the increasing number of patients with extended lifespans after treatment. This study employs immunohistochemistry (IHC) to explore endothelial expressions of E and P selectins, ICAM-1, VCAM-1, eNOS, PAI-1, and MMP-9 in irradiated and non-radiated patients. This study aimed to identify the specific roles of markers in the pathogenesis of cerebral vascular endothelial injury after radiosurgery and explore the distinct expression patterns of each marker in various pathologies following stereotactic radiosurgery (SRS), guiding potential preventive and therapeutic interventions for mitigating long-term radiation adverse effects.

## 2. Methods

A single-center retrospective cohort study analyzed endothelial marker expression through IHC analysis in neurosurgical patients who had undergone SRS from 2001 to 2015 with a follow up duration > 6 months. The study identified distinctive post-radiation vasculopathy in the irradiated vessels and endothelial cells, characterized by edematous and fibrotic alterations in the vascular wall.

**Figure 1.**
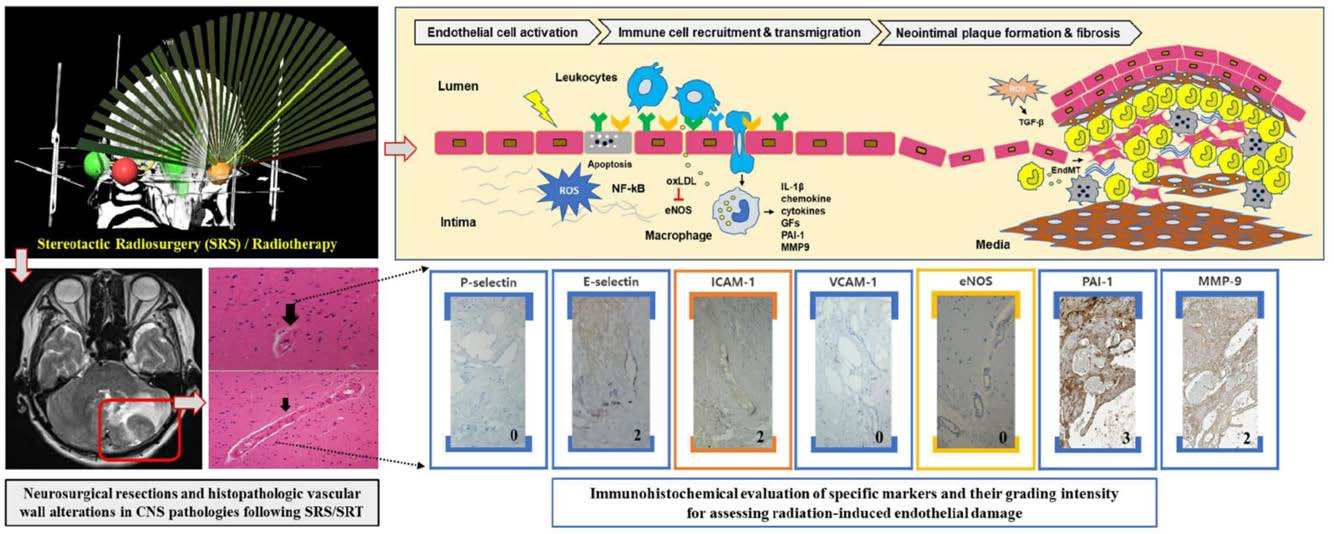
A conceptual graphical abstract illustrating the sequential process of the study. This includes the stages of radiosurgery, sample collection, immunohistochemical analysis, and the investigation of pathophysiological events.

The clinical dataset compiled for this study encompassed detailed radiation doses and an extensive record of post-SRS clinical outcomes, including instances of disease recurrence and radiation-induced adverse effects. The study adhered to ethical standards with Institutional Review Board approval and a waiver for informed consent (IRB No. 2020-07-027-002). Data extraction from the institution electronic medical records was conducted with precision, providing a dependable and precise dataset for subsequent analysis. Figure 1 is a conceptual graphical abstract, visually depicting the process of summarizing the entire study.

### 2.1. Demographic features

Table 1 summarizes the demographic and clinical features of 25 cases, including pathology, diagnosis, radiosurgery timeline, and post-SRS operations. The study comprises 13 males and 12 females, aged 17 to 77 (mean age: 52.2 ± 13.3 years). The Cases were classified into 5 pathologies: cerebral arteriovenous malformations (AVM), glial tumors, meningioma, metastasis, and schwannoma. The average period between SRS and post-SRS surgery was approximately 49.6 ± 49.7 months, ranging from 7.8 to 200.2 months. The average periods between SRS and post-SRS surgery were about 61.2 months, 23.9 months, 101.7 months, 21.9 months, and 53.1 months for cerebral AVM, glial tumors, meningiomas, metastasis, and schwannomas, respectively. The primary reasons for neurosurgery and extraction following radiation surgery is prompted by complications such as adverse effects and edema in cerebral AVM, recurrence in most malignancies, and radiation necrosis in metastatic tumor survivors. For benign tumors like meningiomas or schwannomas, surgery addresses peritumoral edema, localized complications, or the emergence of new lesions. Based on the disease type, the average biologically effective dose (BED) was determined as 212.3 ± 25 Gy_3_ for cerebral AVM. For malignant tumors, including metastatic brain tumors and malignant gliomas, it was either 80 ± 11.3 Gy_10_ or 81 ± 4.5 Gy_10_. For benign tumors such as nerve tumors and meningiomas, the BED was recorded as 63.6 ± 9.8 Gy_3_ or 78.2 ± 5.3 Gy_3_.

**Table 1:** Demographic features.

No.	Sex	Age	Dx	GTV (mm3)	BED (Gy)	Interval (d)	Interval (mo)
1	M	56	Vestibular schwannoma	23,640	49	233	7.8
2	F	67	Metastatic brain tumor	10,090	75.9	635	21.2
3	M	56	Meningioma	39,450	48.9	6006	200.2
4	M	47	Metastatic brain tumor	480	87.5	974	32.5
5	M	62	AVM	3300	183.33	2891	96.4
6	F	38	Hemangiopericytoma	48,525	90	3803	126.8
7	M	39	Glioblastoma	23,424	84	324	10.8
8	F	54	Glioblastoma	66,540	84	678	22.6
9	F	17	AVM	4280	233.33	560	18.7
10	F	43	Meningioma	73	79.3	3932	131.1
11	F	73	Meningioma	34,310	83	416	13.9
12	M	60	Metastatic brain tumor	5020	60	233	7.8
13	F	45	Schwannoma, spinal cord	675	69.33	1634	54.5
14	F	65	Glioblastoma	55,346	84	967	32.2
15	M	36	AVM	4760	233.33	1404	46.8
16	F	77	Meningioma	10,095	90	1097	36.6
17	M	61	Metastatic brain tumor	260	120	600	20.0
18	M	73	Metastatic brain tumor	1561	60	1061	35.4
19	M	70	Metastatic brain tumor	8632	75.9	437	14.6
20	M	52	Glioblastoma	12,360	84	1185	39.5
21	F	49	Oligodendroglioma	111,882	75	674	22.5
22	F	50	Acoustic Schwannoma	20,630	66.67	676	22.5
23	M	46	AVM	13,850	199.33	2485	82.8
24	M	52	Glioblastoma	143,720	75	475	15.8
25	F	53	Schwannoma-NF2	8150	69.33	3826	127.5

No = number of cases, Dx = diagnosis, GTV = gross target volume, BED = biologically effective dose, interval = period of surgery after radiosurgery.

### 2.2. Immunohistochemical staining

Arterial vessel samples were procured from 25 irradiated patients with prior cranial SRS and various pathologic diagnoses. IHC staining was performed by Ventana Bechmark XT machine system. Serial sections (4 μm thick) of formalin-fixed, paraffin-embedded samples were cut and mounted on glass slides. Sections were dewaxed by passage through xylene and then rehydrated in graded alcohol (100%, 95%, and 75%). The sliced tissues were transferred to 1 mM EDTA, pH 8.0 for 30 minutes to retrieve antigens for P-selectin, E-selectin, ICAM1, VCAM1, eNOS, PAI-1, and MMP9 staining. After washing in water, 3.0% H2O2 in methanol was applied for 20 minutes in order to block endogenous peroxidase activity, and the slides were incubated at room temperature for 20 minutes. Next, the slides were incubated at 4°C overnight with a primary antibody against P-selectin (1:100; abcam ab118522), E-selectin (1:100; abcam ab18981), ICAM1(1:400; Santa Cruz G-5), VCAM1(1:400; abcam EPR5047), eNOS (1:500; abcam ab5589), PAI-1(1:250; Santa Cruz C-9), and MMP9 (1:250; abcam ab38898) staining for 32 minutes. Tissue was stained using an ultraView Universal DAB Detection Kit from Ventana and counterstained with Mayer hematoxylin. Negative control sections were stained under identical conditions as the buffer solution substituting for the primary antibody. The tissues underwent staining for the markers, with intensity and extent of positivity being meticulously evaluated. Intensity was scored using a 4-tier grading system: 0 (negative), 1 + (mild), 2 + (moderate), and 3 + (strong). Normal control tissues were obtained from non-neoplastic brain parenchyma contained in cerebrovascular hematoma removal tissue.

### 2.3. Statistical analysis

The statistical analysis aimed to evaluate IHC expressions and identify any significant differences in the cumulative sequential expression of specific markers, which are pivotal in the development of vasculopathy using a Two-Way Analysis of Variance (ANOVA). Additionally, to discern the disparities in staining grades among different diseases, the Kruskal–Wallis test was utilized. The data were analyzed using GraphPad Prism version 10 (GraphPad Software, San Diego, CA, USA), ensuring robust and reliable statistical processing. The results of these analyses are presented in the form of plot charts, which clearly illustrate the mean values alongside their standard deviations, providing a visual representation of the data distribution and variability among the groups.

## 3. Results

### 3.1. Individual marker expression and cumulative IHC values across different pathologies

**Figure 2.**
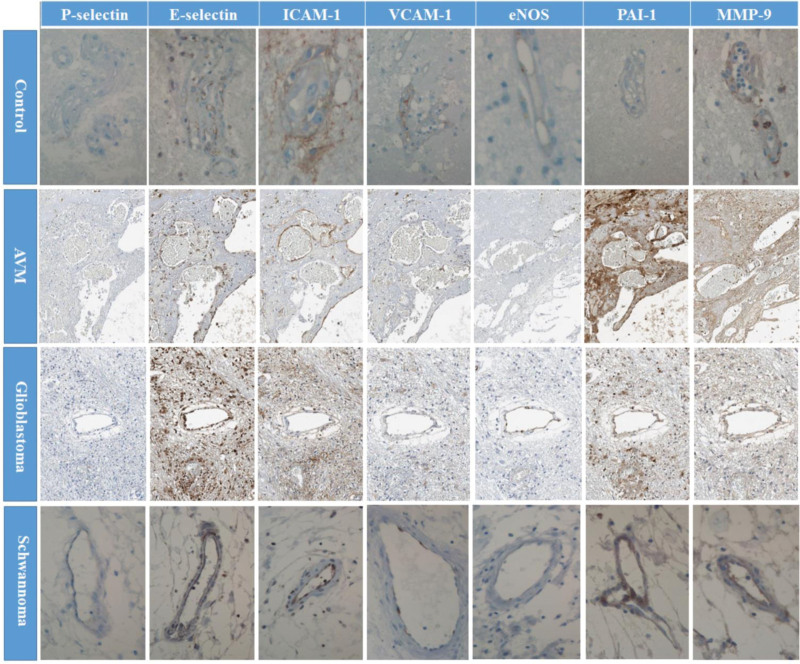
Demonstration of variety of marker expression by immunohistochemical staining in control tissues and pathological samples. The control group shows low levels (grade 1) of ICAM-1, VCAM-1, and MMP-9, with no expression (grade 0) of P-selectin, E-selectin, eNOS, and PAI-1. For the pathological samples: P-selectin was not expressed; E-selectin had moderate expression in AVM and schwannoma, with strong expression in glioblastoma; ICAM-1 was weakly expressed in glioblastoma, yet strongly expressed in AVM and schwannoma; VCAM-1 showed weak expression across all samples; eNOS was not expressed in AVM and schwannoma but was strongly expressed in glioblastoma; PAI-1 had moderate expression in glioblastoma and schwannoma, with strong expression in AVM; and MMP-9 showed moderate expression in all pathological conditions. Magnification x200 for all images.

The tissue sections from a total of 25 cases were assessed to evaluate the expression of specific markers associated with various neurosurgical pathologies using IHC. Immunohistochemistry provided a visual means to identify and quantify the presence and levels of targeted proteins within the tissue samples, employing a grading scale from 0 to 3. The individual marker expression was detailed for various cases, including control, AVMs, malignant tumors such as glial tumors and metastatic brain tumors, and benign tumors including meningiomas and schwannomas. Immunohistochemical staining for 7 markers in 4 representative case studies is depicted in Figure 2. The summarized findings are as follow: In the control group, E-selectin, ICAM-1, and MMP-9 each demonstrated a grade 1 expression, while no staining (grade 0) was observed for the other markers.

For the AVM specimen (Case 15), E-selectin and ICAM-1 were markedly expressed, with staining grades of 2 and 3, respectively. The markers VCAM-1, PAI-1, and MMP-9 showed mild to moderate staining, with respective grades of 1, 2, and 2. In contrast, P-selectin and eNOS did not exhibit any staining. In the glioblastoma sample (Case 24), pronounced expression was observed for E-selectin and eNOS, each with a staining intensity of grade 3. ICAM-1 and VCAM-1 displayed mild staining with a grade of 1, while PAI-1 and MMP-9 each had a staining grade of 2. The schwannoma specimen (Case 25) was characterized by a particularly strong expression of ICAM-1 with a staining grade of 3. VCAM-1 exhibited milder staining with a grade of 1. Moderate staining was observed for E-selectin, PAI-1, and MMP-9, with each marked as grade 2. eNOS staining was absent (0). A notable trend across all the samples was the consistent lack of P-selectin staining. Moreover, PAI-1 and MMP-9 expressions were consistently observed with grades above 2 in the pathologies detailed in Figure 2.

Table 2 compiles the average IHC expressions with their standard deviations for these markers across the different pathologies. The values are presented as follows: P-selectin (0.24 ± 0.74), E-selectin (2.04 ± 0.89), ICAM-1 (2.20 ± 0.76), VCAM-1 (0.56 ± 0.92), eNOS (0.73 ± 0.98), PAI-1 (1.20 ± 1.12), and MMP-9 (1.32 ± 0.95). The median cumulative IHC expression levels across the various pathologies were observed as AVM (1.50), Glial tumors (1.83), Meningioma (0.40), Metastasis (1.00), and Schwannoma (1.25), respectively. Upon statistical analysis using the Kruskal–Wallis test, no significant differences were found in the IHC expression patterns across the pathologies (*P* = .53).

**Table 2:** Summary of average IHC expression of the individual marker throughout various pathological groups (average ± SD).

Pathology	P-selectin (25)	E-selectin (25)	ICAM1 (25)	VCAM1 (25)	eNOS (25)	PAI-1 (25)	MMP9 (25)	Median
AVM (4)	0.5 ± 1.0	2.0 ± 1.4	3.0 ± 0.0	0.25 ± 0.5	0.8 ± 1.0	1.5 ± 1.3	2.0 ± 0.8	1.50
Glial tumors (6)	0.0 ± 1.0	2.2 ± 0.8	1.8 ± 0.8	0.7 ± 0.8	1.2 ± 1.2	1.8 ± 0.8	1.8 ± 0.8	1.83
Meningioma (5)	0.0 ± 0.0	1.8 ± 0.5	2.0 ± 0.7	0.2 ± 0.5	0.0 ± 0.0	0.4 ± 0.6	1.0 ± 0.0	0.4
Metastasis (6)	0.5 ± 1.2	2.0 ± 1.1	2.0 ± 0.6	0.25 ± 0.5	0.5 ± 1.3	1.0 ± 1.6	0.7 ± 0.8	1.0
Schwannoma (4)	0.3 ± 0.5	1.5 ± 1.3	3.0 ± 0.0	1.3 ± 1.3	0.3 ± 0.5	1.0 ± 1.2	1.5 ± 1.3	1.25
Mean (25)	0.24	2.04	2.20	0.56	0.728	1.20	1.32	-
Std. Deviation	0.74	0.89	0.76	0.92	0.98	1.12	0.95	-
Control	0	1	1	1	0	0	1	-

() = number of cases, Std. Deviation = standard deviation.

### 3.2. Comparative IHC expression of markers across the pathologies

**Figure 3.**
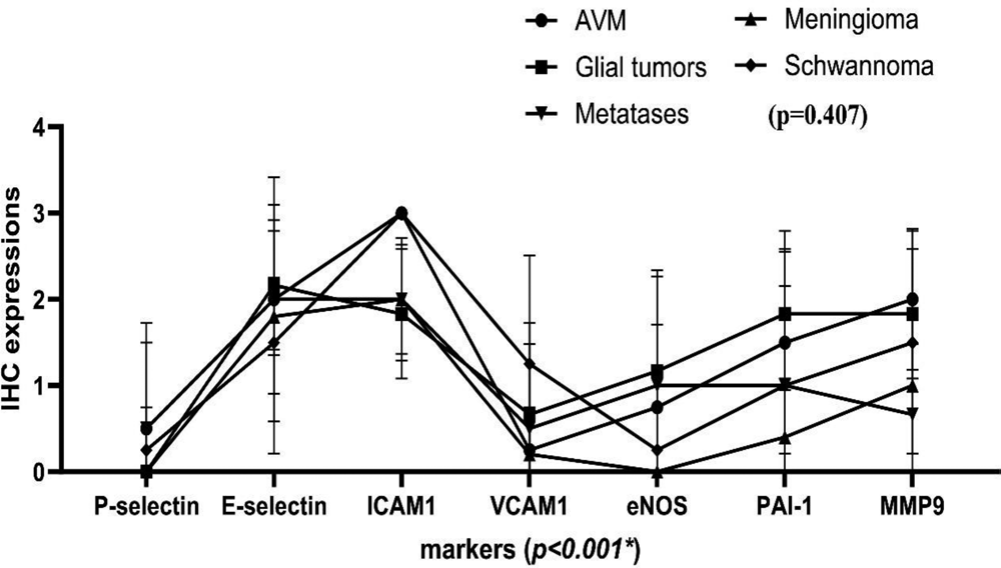
Immunohistochemistry expressions of 7 markers across the different pathologies. Notably, while individual markers showed significant variations in expression levels when comparing different diseases (*P* < .001), no substantial differences were found in the overall IHC expression patterns across the 5 distinct pathologies studied (*P* = .4070, via 2-way ANOVA).

The IHC expression levels of individual markers were analyzed and compared across various pathologies, as shown in Figure 3. The following details the expression profiles for each pathology:

AVMs Group: ICAM-1 showed strong average IHC expression, followed by more moderate expression levels of E-Selectin, MMP-9, and PAI-1. P-Selectin, eNOS, and VCAM-1 were minimally expressed.

Glial Tumors Group: Notably, E-Selectin, ICAM-1, PAI-1, MMP-9, and eNOS were expressed predominantly, whereas VCAM-1 showed lower expression, and P-Selectin was not expressed.

Meningiomas Group: ICAM-1, E-Selectin, and MMP-9 were the most expressed markers, while PAI-1 and VCAM-1 exhibited less expression. Both P-Selectin and eNOS had no expression.

Metastases Group: E-Selectin, ICAM-1, and PAI-1 displayed prominent IHC expression. In contrast, MMP-9, P-Selectin, eNOS, and VCAM-1 had relatively lower expression levels.

Schwannomas Group: ICAM-1 was the most expressed, with E-Selectin, MMP-9, PAI-1, and VCAM-1 showing moderate expression. In contrast, eNOS and P-Selectin were least expressed.

A comprehensive analysis of the cumulative IHC expression across pathologies highlighted significant variability in marker expression (*P* < .001, with an F (6, 120) = 24.90, 2-way ANOVA), as shown in Figure 3. These results indicated marked differences in the expression levels of the markers when considering all pathologies together. High Levels of Expression: ICAM-1 and E-selectin were found to have consistently high levels of IHC expression across the pathologies studied. Moderate and Low Levels of Expression: MMP-9, PAI-1, and eNOS were expressed at moderate levels, and VCAM-1 and P-selectin had the least expression. Despite these variations in individual marker expression, our findings did not reveal any statistically significant differences in the overall patterns of IHC expression when comparing the 5 distinct pathologies (*P* = .407, F (4, 20) = 1.049, 2-way ANOVA, Figure 3).

### 3.3. Overview of cumulative IHC expressions

**Figure 4.**
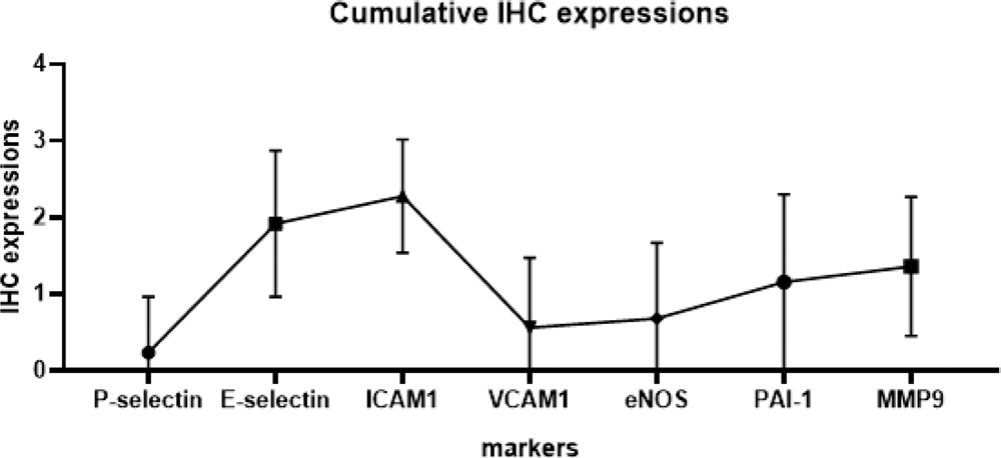
Cumulative immunohistochemistry expression trends based on the markers. The sequential IHC expression trends are visualized, with statistical analysis indicating significant findings (*P* < .001, ANOVA), which underscore the progression and intensity of marker expression over time.

The comparative analysis of IHC expression across studied pathologies indicated that ICAM-1 and E-selectin consistently exhibited strong IHC expression. Moderate expression levels were noted for MMP-9, PAI-1, and eNOS, whereas VCAM-1 and P-selectin presented the least expression within the study. Upon conducting a cumulative IHC expression analysis, significant variations in marker expressions were identified (*P* < .001, F (24, 144) = 3.524), as delineated in Figure 4 in which findings underscore substantial differences in marker expression across the broad range of investigated pathologies.

### 3.4. IHC expression aligned with vasculopathy cascade sequence

**Figure 5.**
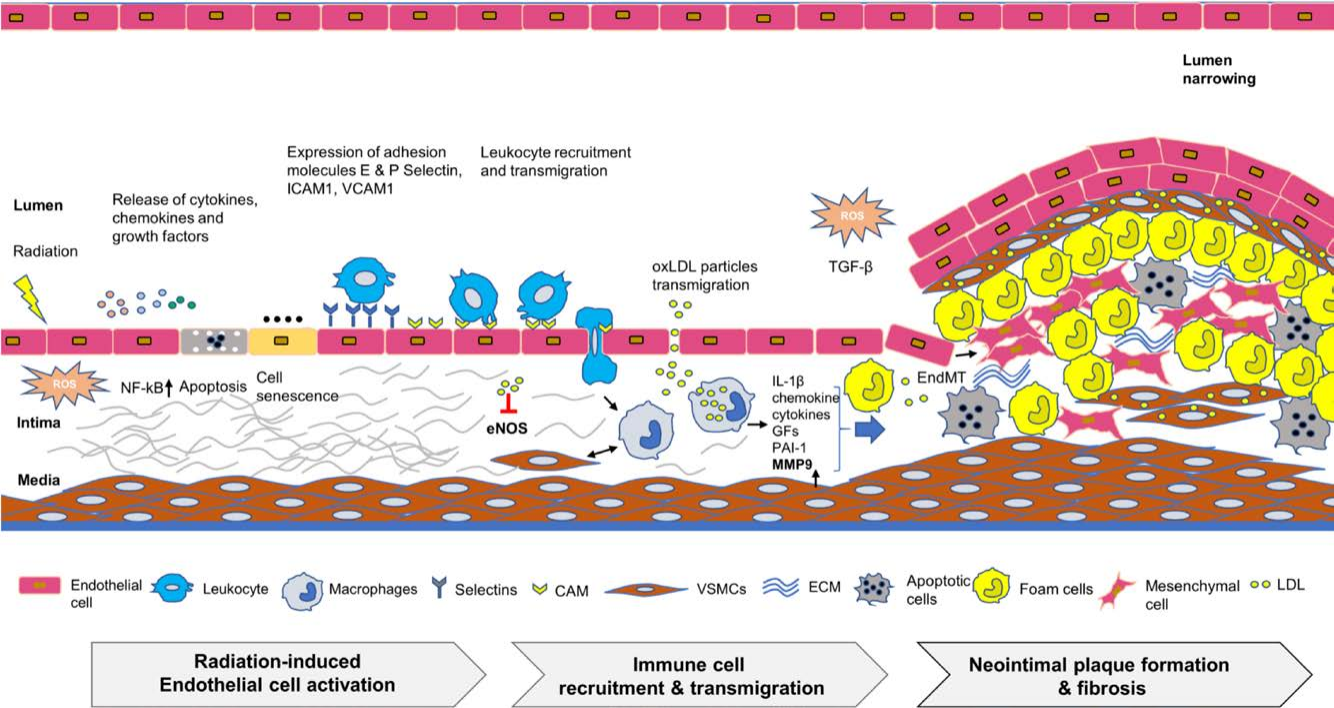
Schematic of the Pathogenesis of Radiation-Induced Vascular Response: A detailed diagram depicting the sequence of events in the pathogenesis of radiation-induced vascular injury, from the initial endothelial damage to the final stages of fibrosis and neovascularization. CAM = cell adhesion molecule, ECM = extracellular matrix, EndMT = endothelial mesenchymal transformation, IL-1β = interleukin-1β, MMP9 = matrix metalloproteases-9, oxLDL = oxidized low-density lipoprotein, ROS = reactive oxygen species, TGF-β = transforming growth factor-β, VEGF = vascular endothelial growth, VSMC = vascular smooth muscle cell.

In the context of endothelial dysfunction and leukocyte transmigration into the intimal layer, ICAM-1 and E-selectin were consistently expressed at significant levels. Reduced eNOS expression aligns with the stage where oxidized low-density lipoprotein (LDL) suppresses eNOS activity, potentially contributing to atherosclerotic plaque development. Conversely, ICAM-1 and P-selectin expression levels were diminished. Pronounced expression of MMP-9 and PAI-1, indicative of neovascularization and vascular fibrotic lesion, was observed across all samples (Fig. 5).

## 4. Discussion

Radiotherapy detrimentally affects the vascular endothelium, with proposed mechanisms involving endothelial dysfunction, inflammation, mitochondrial dysfunction, and vascular barrier disruption mediated by specific cytokines and adhesions molecules.^[6]^ This section briefly elucidates the pathogenic mechanisms of radiation-induced vascular sequelae, emphasizing the study significant findings.

### 4.1. Endothelial activation and dysfunction

Radiation activates endothelial cells directly or indirectly through pathways like ROS generation, cytokine release, and angiogenic disruption. Radiation-induced ROS compromise cellular macromolecules, inducing apoptosis and senescence.^[7]^ Endothelial senescence, particularly due to post-radiation DNA damage, fosters a pro-inflammatory and pro-thrombotic environment, catalyzing atherogenesis.^[10,17]^ ROS activate the nuclear factor-kappa B (NF-kB) pathway, enhancing expression of thrombosis and inflammation markers.^[11]^ Radiation-induced molecules (VCAM-1, ICAM-1, E-selectin) worsen endothelial dysfunction by attracting and activating immune cells, perpetuating inflammation. Additionally, eNOS uncoupling produces ROS, contributing to diseases like atherosclerosis, promoting thrombogenesis and atherogenesis.^[9]^

### 4.2. Inflammation and leukocyte transmigration

Leukocyte recruitment encompasses stages including tethering, rolling, arrest, adhesion, and transmigration.^[13]^ Recruitment starts with leukocytes tethering to the endothelium, facilitated by P- or E-selectin, progressing to slow rolling and firm arrest.^[9]^ At this stage, ICAM-1 bolsters leukocyte rolling, adhesion, and extravasation.^[18]^ Leukocyte-endothelial adhesion marks the onset of atherogenesis.^[9]^ Activating factors, such as cytokines, stimulate these leukocytes.^[13]^ Moreover, MMP-9 facilitates monocyte trans-endothelial migration by disrupting the adjacent basement membrane.^[12]^ Intimal oxidized lipid uptake by macrophages spurs primary atherosclerotic lesion development, inducing subsequent inflammatory responses, thereby leading to atherosclerosis progression,^[16]^ (Fig. 5).

### 4.3. Plaque formation and thrombosis

Factors contributing to atherosclerotic plaque formation and necrotic core development include interleukin-1β (IL-1β) secretion, vascular smooth muscle transdifferentiation, and leukocyte accumulation (Fig. 5). Chemokines, cytokines, and growth factors further enhance fibromuscular plaque development.^[14]^ PAI-1 may stimulate neointimal plaque formation, increasing thrombosis risk.^[15]^ Furthermore, oxidized LDL downregulates eNOS expression, detrimentally affecting endothelial cells and promoting plaque progression.^[19]^ DNA damage-induced cell senescence caused by radiation injury may reinforce fibrosis through pro-inflammatory factors, transforming growth factor-β (TGF-β) signaling, and fibroblast activity.^[20]^ Clinically, plaque rupture is concerning as it often precedes thrombosis.^[21]^

### 4.4. Angiogenesis and neovascularization

In vascular atherosclerotic lesions, radiation induced neovascularization enhances plaque growth, instability, and rupture through the contributing factors including ROS, inflammation, oxidized lipids, and metalloproteases like MMP-9.^[22]^ Vascular smooth muscle cells (VSMCs) also secrete vascular endothelial growth factor (VEGF), vital for neovascularization and linked to atherosclerotic plaque destabilization.^[23]^ Intraplaque hypoxia, a potent angiogenesis stimulator, can exacerbate lesions, induce intraplaque hemorrhage, and amplify oxidative and inflammatory responses.^[16]^

### 4.5. Endothelial-to-Mesenchymal Transition (EndMT)

Endothelial cells transition to a mesenchymal phenotype occurs via pathways like TGF-β, Notch, and Wnt/β-catenin, influenced by factors like oxidative stress, metabolic changes, and hypoxia (Fig. 5). Resultant mesenchymal cells can differentiate into various cell types, including fibroblasts and myofibroblasts. Endothelial-to-Mesenchymal Transition, implicated in diseases like fibrosis and atherosclerosis, is a potential therapeutic target.^[24]^ Prior research highlights differences in endothelial marker expression after radiation, complicating the understanding of radiation-induced vascular changes.^[25]^ Increased ICAM-1, E-selectin, and PAI-1 align with our findings, emphasizing their importance in early vascular damage. However, inconsistencies, such as the elevated P-selectin and VCAM-1 not aligning with our results, may stem from variations in radiation dose, tissue sensitivity, exposure time, and individual susceptibilities.^[1,25,26]^ Our study highlights key findings regarding the expression of endothelial markers in radiation-induced vascular damage. Elevated ICAM-1 and E-selectin levels across all cases emphasize their crucial role in the early phases of vascular damage, while MMP-9 and PAI-1 become more relevant in later stages, correlating with vascular complications and showcasing the dynamic nature of these processes.

Analyzing immunohistochemical patterns across different pathologies after radiation therapy reveals valuable insights. In AVM, strong ICAM-1 expression suggests an inflammatory-driven pathology,^[27–29]^ whereas glial tumors exhibit low IHC expression and the absence of P-selectin, indicating minimal prothrombotic involvement.^[6,27,29–31]^ Meningioma and metastasis groups, with varied IHC expressions, suggest a lower likelihood of thrombotic events but a consistent inflammatory pathogenesis.^[27]^

In metastatic tumors, moderate expressions of E-selectin and ICAM-1 suggest an inflammatory-driven pathogenesis, with reduced likelihood of thrombotic processes.^[6,32]^ Notably, markers involved in immune cell recruitment to injury sites show moderate expression in reactive gliosis, supporting the hypothesis that inflammation is a significant consequence of radiation exposure.^[6,13,25,27,29,30]^ This inflammatory pattern is further substantiated in the schwannoma group, where substantial ICAM-1 expression indicates a predominant inflammatory mechanism following radiation therapy.^[25,27–29]^ Despite variations in pathologies, consistent findings across different cases suggest that, while prothrombotic processes are present, the inflammatory reaction triggered by radiation predominantly influences the progression of these conditions. Overall, radiation-induced vascular changes are traceable through the differential expression of these markers, serving as indicators of the pathology stage and nature. Early alterations involve increased expression of adhesion molecules (ICAM-1 and E-selectin), facilitating immune cell dynamics, while advanced stages with neovascularization and thrombosis within plaques manifest with pronounced MMP-9 and PAI-1 expression. Intriguingly, regardless of the disease type, radiation exposure consistently induces a pattern of marker expression, revealing a commonality in the vascular response to radiation stress. This study, however, acknowledges the limitation in evaluating all markers pivotal to understanding the full spectrum of radiotherapy-induced vasculopathy.

## 5. Conclusion

The initial response to radiation-induced vascular injury involves increased adhesion molecules such as ICAM-1 and E-selectin. These molecules play a crucial role in recruiting immune cells to inflamed endothelium, facilitating their transmigration into the sub-endothelium of the intimal layer. As the condition progresses, later stages are marked by the development of new blood vessels within plaques, accompanied by fibrosis and clot formation, correlating with elevated levels of MMP-9 and PAI-1.

Despite the diverse long-term effects of radiotherapy on the vascular endothelium, a common theme of inflammation underlies the pathology of these conditions. The distinct patterns of marker expression identified in our study suggest that different markers play unique roles in the development of radiation-induced vasculopathy. These findings provide valuable insights that could pave the way for the development of innovative preventive strategies and treatments.

## Author contributions

**Conceptualization:** Moon-Jun Sohn.

**Data curation:** Mohammad Mohsen Mosleh, Moon-Jun Sohn, Han Seong Kim.

**Formal analysis:** Mohammad Mohsen Mosleh, Moon-Jun Sohn.

**Funding acquisition:** Moon-Jun Sohn.

**Investigation:** Mohammad Mohsen Mosleh, Han Seong Kim.

**Methodology:** Moon-Jun Sohn, Han Seong Kim.

**Software:** Mohammad Mohsen Mosleh.

**Supervision:** Moon-Jun Sohn.

**Visualization:** Han Seong Kim.

**Writing – original draft:** Mohammad Mohsen Mosleh.

**Writing – review & editing:** Mohammad Mohsen Mosleh, Moon-Jun Sohn, Han Seong Kim.
